# Eco-Evolutionary Effects of Bacterial Cooperation on Phage Therapy: An Unknown Risk?

**DOI:** 10.3389/fmicb.2020.590294

**Published:** 2020-11-12

**Authors:** Adrián Cazares, Rodolfo García-Contreras, Judith Pérez-Velázquez

**Affiliations:** ^1^EMBL’s European Bioinformatics Institute (EMBL-EBI), Wellcome Genome Campus, Cambridge, United Kingdom; ^2^Wellcome Sanger Institute, Wellcome Genome Campus, Cambridge, United Kingdom; ^3^Department of Microbiology and Parasitology, Faculty of Medicine, Universidad Nacional Autónoma de México, Mexico City, Mexico; ^4^Zentrum Mathematik, Technical University of Munich, Garching, Germany; ^5^Technische Hochschule Ingolstadt, Institute of Innovative Mobility, Ingolstadt, Germany

**Keywords:** phages, phage therapy, quorum sensing, bacteria-phage interactions, cheaters, anti-phage defense

## Abstract

If there is something we have learned from the antibiotic era, it is that indiscriminate use of a therapeutic agent without a clear understanding of its long-term evolutionary impact can have enormous health repercussions. This knowledge is particularly relevant when the therapeutic agents are remarkably adaptable and diverse biological entities capable of a plethora of interactions, most of which remain largely unexplored. Although phage therapy (PT) undoubtedly holds the potential to save lives, its current efficacy in case studies recalls the golden era of antibiotics, when these compounds were highly effective and the possibility of them becoming ineffective seemed remote. Safe PT schemes depend on our understanding of how phages interact with, and evolve in, highly complex environments. Here, we summarize and review emerging evidence in a commonly overlooked theme in PT: bacteria-phage interactions. In particular, we discuss the influence of quorum sensing (QS) on phage susceptibility, the consequent role of phages in modulating bacterial cooperation, and the potential implications of this relationship in PT, including how we can use this knowledge to inform PT strategies. We highlight that the influence of QS on phage susceptibility seems to be widespread but can have contrasting outcomes depending on the bacterial host, underscoring the need to thoroughly characterize this link in various bacterial models. Furthermore, we encourage researchers to exploit competition experiments, experimental evolution, and mathematical modeling to explore this relationship further in relevant infection models. Finally, we emphasize that long-term PT success requires research on phage ecology and evolution to inform the design of optimal therapeutic schemes.

## Introduction

In some bacteria, such as *Vibrio cholerae*, phage defense mechanisms are induced by quorum sensing (QS), whereas in others, such as *Pseudomonas aeruginosa*, QS promotes phage susceptibility. Whether bacteria use QS to repress or promote virulence, increasing evidence points to a close connection between QS and modulation of susceptibility to phage infection. The perspective presented here aims to briefly revisit phage therapy (PT) in view of recently reported phage-host interactions, summarize emerging evidence on the QS-phage susceptibility relationship, discuss potential implications in the context of PT, and explore how this knowledge may help to develop antibacterial strategies that reduce the risk of undesired side effects.

## Revisiting Phage Therapy

The idea of using bacteriophages to treat bacterial infections, namely, PT, is as old as phage discovery itself, however, the introduction and widespread use of antibiotics, among other factors, limited further PT development on a global scale (Casey et al., 2018). Given the current antibiotic crisis, PT is facing a prominent second wave of interest supported by successful case reports (Sarker et al., 2016; Chan et al., 2018; Duplessis, 2018; Furr et al., 2018; LaVergne et al., 2018; Dedrick, 2019) and creation of PT-oriented research centers and private biotech companies (Adaptive-Phage-Therapeutics, n.d.; Center for Innovative Phage Applications and Therapeutics, n.d.; PhagoMed, n.d.). This year, the use of bacteriophages was proposed as a potential “game changer” in the efforts to reduce the mortality rate in patients infected with the SARS-Cov-2 virus, and particularly in those developing secondary bacterial infections (Wojewodzic, 2020), whereas other research work reported the potential use of phage-derived enzymes in controlling the emergence of intestinal pathobionts (Fujimoto et al., 2020). Notably, these reports reflect the current enthusiasm for applying PT on a large scale and in a broad variety of health problems.

As PT advances toward becoming a true medical alternative, questions naturally arise about potential limitations and negative effects of using phages as antimicrobials. Determinants limiting phage infection (e.g. spatial structure of the infection environment and microbial community diversity), potential incompatibility with other therapies, difficulties on the production and stability of phage stocks, safety and side effects issues, represent factors impeding further PT development (Oechslin, 2018; Torres-Barceló, 2018; Breederveld, 2019). Importantly, several of these factors can be linked to our lack of knowledge about the ecological and evolutionary impact of PT in an ecosystem consisting not only of the therapeutic phage and its target pathogen but also the host microbiome and immune system.

The coexistence of multiple biotic and abiotic elements at the site of infection implies a complex network with a plethora of interactions about which very little is currently known. The effect of phages on the immune system and selection of resistance to phage infection stand as some of the major PT concerns resulting from this interaction network. As recent research shows, phage interaction with the immune system is intricate and can subvert immune response (Sweere et al., 2019). Filamentous phages infecting *P. aeruginosa* can physically interact with mammalian immune cells triggering a maladaptive immune response that results in impaired bacterial clearance during infections (Sweere et al., 2019). These phages have been also involved in an immune response impairing keratinocyte migration and leading to delayed healing of *P. aeruginosa*-infected wounds (Bach et al., 2020). Although filamentous phages do not represent a virus type selected for therapy purposes, these studies highlight the complex consequences of previously unknown interactions between phages and the immune system. Furthermore, this interaction is not restricted to filamentous phages, since viruses of the order Caudovirales, typically selected for PT, can also directly interact with immune systems, stimulating a response that exacerbates intestinal inflammation and colitis (Bollyky, 2019; Gogokhia et al., 2019).

In recent years, progress has also been made on the understanding of how bacteria evolve cross-resistance against multiple phages. For example, the comprehensive characterization of a bacteria-phage interaction network indicated that cross-resistance is common and associated with different genetic basis related to exposure to distinct phages (Wright et al., 2018). Yet, the study showed that mutations in diverse phage receptors structure the modularity of the network and feature lower fitness cost than other mutations in regulatory genes driving more generalist phage resistance (Wright et al., 2018). Further research revealed that timing and order of phage exposure are factors that shape the evolutionary trajectory of cross-resistance as they are associated with a different mutational basis, strength of resistance, and fitness cost (Wright et al., 2019). These studies underscore the intricate nature of phage-bacteria interactions and their impact in the emergence of key evolutionary traits such as phage resistance, a factor that should be considered in the design of long-term PT strategies.

We seem to be on the brink of using PT on a larger scale, therefore, it is pivotal to start addressing how the interaction of phages with elements of the surrounding environment, especially in the context of infections, can impact PT outcomes. Here, we focus on the implications of a so far overlooked interaction with a profound effect in the biology of bacteria and their viruses: phages and cell-cell communication or QS. Emerging evidence reveals that this interrelationship is very complex and multidimensional since QS can regulate various anti-phage defense mechanisms, whereas phages can influence cooperative behaviors and hack QS systems to mediate their own gene expression. Phage-associated intercellular communication will not be discussed in this perspective but it has been recently addressed by Igler and Abedon (2019).

## Quorum Sensing, Cooperation, and Cheating

QS is a prevalent mechanism for gene expression regulation in bacterial populations *via* self-produced and diffusible signal molecules (Schuster et al., 2017). QS primarily coordinates cooperative behavior (e.g., virulence and nutrient digestion) through the secretion of extracellular products such as toxins, exopolysaccharides, or enzymes (Schuster et al., 2017). These secreted products are considered public goods since the population benefits from them in a cell density-dependent manner. Importantly, besides public goods, QS can control the production of traits that only benefit the producer individual, hence referred to as private goods (Schuster et al., 2017).

QS-mediated cooperation is a key population attribute; however, its maintenance is often challenged by the emergence of individuals that profit from public goods without contributing to their production, mutants commonly known as cheaters. Exploitation by cheaters can ultimately lead to population collapse but several factors preventing this scenario have been reported, including the action of QS-controlled private goods that stabilize cooperation (Dandekar et al., 2012; García-Contreras et al., 2014). In this context, preferential phage infection toward a subpopulation, namely producers or cheaters, typically represented by wild-type or mutant individuals, can define the ecological and evolutionary fate of the population. This situation may occur in infections where the emergence of QS mutants has been documented. *P. aeruginosa* (Hoffman et al., 2009; Wang et al., 2018) and *Staphylococcus aureus* (Paulander et al., 2012) represent examples of pathogens for which the occurrence of QS mutants has been reported in the clinic. Therefore, investigating the effect of QS on phage susceptibility is relevant to anticipate PT outcomes, especially considering that QS is commonly involved in virulence regulation and selecting one subpopulation could derive unintended consequences.

## Quorum Sensing and Anti-Phage Defense Mechanisms

A growing body of evidence implicates QS in regulating anti-phage defense strategies. In some instances, QS coordinates downregulation of diverse cell membrane proteins acting as phage receptors, thus resulting in reduced phage susceptibility. This is the case for the receptor LamB in *Escherichia coli* (Høyland-Kroghsbo, 2013), OmpK in *Vibrio anguillarum* (Tan et al., 2015), and O1 in *V. cholerae* (Hoque, 2016). In *E. coli*, QS controls the concomitant downregulation of multiple receptors, including the flagellum, thus widening the range of protection against phage infection. Likewise, in *V. cholerae*, QS is additionally associated with increased emergence of phage resistant individuals and production of hemagglutinin protease, a protein causing extracellular phage inactivation (Hoque, 2016). More recently, QS was also linked to promotion of extracellular proteolytic activity affecting the virion stability of different phages in *V. anguillarum* (Tan et al., 2020). In this study, the authors also report the association between QS and reduction of H20-like prophage induction (Tan et al., 2020), adding another layer of complexity to the phage-bacteria interactions *via* density-dependent cell-cell communication.

*P. aeruginosa* represents another example where QS has been associated with reduction in plaque production, burst size, and expression of the phage RNA polymerase (Qin et al., 2017). Although the mechanism behind this response has yet to be deciphered, it seems to be independent of phage adsorption and related to an increase in the proportion of dormant cells (Qin et al., 2017). It is worth noting, however, that this response appears to be strain-dependent, as other studies in *P. aeruginosa* show a different QS-phage infection relationship (see below). Activation of the adaptive immune system CRISPR-Cas has been also associated with QS (Høyland-Kroghsbo et al., 2017), however, it is unclear to what extent a deficient QS system would impact phage susceptibility *via* this mechanism.

The link between QS and anti-phage defense is consistent with the increased risk in spread of infection at higher cell densities, but also implies a strong role for phages in mediating intra-population bacterial competition. In most of the cases described thus far, QS-mediated defense mechanisms provide a private benefit to the producers’ subpopulation (e.g., lower receptor expression or metabolic activity), but at least two examples correspond to anti-phage protection as a public good (hemagglutinin protease production and promotion of extracellular proteolytic activity). In the context of PT, the theory suggests that QS-controlled phage tolerance as a private good could lead to selection of the producer’s subpopulation, in view of their lower susceptibility to phage infection compared to the cheater individuals. On the other hand, phage defense in the form of a public good portrays a more complex scenario because it would be prone to cheating, as we discuss below.

Remarkably, the QS-phage infection interlink is not unidirectional, since QS has also been connected with increased phage susceptibility. In *E. coli* (Taj et al., 2014) and *P. aeruginosa* (Glessner et al., 1999; Mumford and Friman, 2017; Saucedo-Mora et al., 2017), QS has been linked with higher infection rate, represented by expanded plaque production, plaque size, or overall lytic activity. A role of QS in the assembly of type 4 pili, a phage receptor involved in cell twitching motility, was hypothesized to be the cause of increased phage susceptibility in *P. aeruginosa* (Glessner et al., 1999). Although further research uncoupled the direct link between QS and twitching motility, the study revealed that QS mutants readily accumulate secondary mutations associated with loss of twitching motility (Beatson et al., 2002). Other mechanisms underlying the QS-promoted phage susceptibility in *P. aeruginosa* and *E. coli* remain largely unknown. This alternative side of the relationship between QS and phage susceptibility also has potential implications in the PT context: in principle, phages could infect producer individuals preferentially, ultimately selecting the cheaters subpopulation over time. Such selection could have positive consequences in terms of virulence in pathogens where this trait is controlled by QS; nevertheless, other factors may help to mitigate the strength of selection given the importance of QS in regulating multiple cooperative attributes in the population. In Table 1, we compile examples of QS-Phage interactions and briefly describe hypothetical scenarios about their impact on intrapopulation competition and PT outcomes.

**Table 1:** Influence of quorum sensing on phage susceptibility

QS reduces phage susceptibility					
Phage	Type	Host	Reference	Potential effect in intra-population competition	Hypothetical impact in PT
KVP40	Virulent	*V. anguillarum*	Tan et al., 2015	In a mixture of wild-type and QS-deficient bacteria, the phage will infect the mutant phenotype preferentially as a result of the higher phage receptor expression in the QS-deficient cells, thus resulting in selection of the wild-type phenotype over time.	In cases where QS positively regulates bacterial virulence factors, PT could lead to increased virulence: if phages fail to eradicate the target bacterial population, the surviving individuals will most likely correspond to virulent wild-type cells that can then proliferate at the site of infection.
Various (15)	Virulent	*V. cholerae*	Hoque, 2016		
Lambda, X	Temperate, Unknown	*E. coli*	Høyland-Kroghsbo, 2013		
K5, C11	Virulent	*P. aeruginosa*	Qin et al., 2017	In a mixture of wild-type and QS-deficient bacteria, the phage will be more successful at infecting the mutant individuals due to the lower metabolic activity of the wild-type strain, thus promoting the selection of the wild-type phenotype over time.	
**QS increases phage susceptibility**					
**Phage**	**Type**	**Bacterium**	**Reference**	**Potential effect in intra-population competition**	**Hypothetical impact in PT**
D3112cts	Temperate	*P. aeruginosa*	Glessner et al., 1999	Phage will attack the wild-type population at a higher rate due to higher expression of the phage receptor	If PT fails to eradicate bacteria, it will potentially select a sub-population with low virulence and prone to be eliminated by the immune system.
D3112, JBD30	Temperate	*P. aeruginosa*	Saucedo-Mora et al., 2017	Phages infect the wild-type strain more efficiently, however, in a mixture of wild-type and QS-deficient bacteria, phages select the wild-type phenotype, likely resulting from more efficient lysogenesis in the wild-type population leading to fitness increase	If PT fails to eradicate bacteria, it will potentially select the wild-type phenotype, increasing virulence
T4	Virulent	*E. coli*	Taj et al., 2014	In a mixture of wild-type cells and mutants unable to detect QS signals, the phage will exhibit higher lytic activity in wild-type individuals if acyl-homoserine lactone signaling molecules are present in the medium. In contrast, in presence of the indole signal, the phage will reduce its lytic activity in cells able to detect this molecule. Therefore, selection will depend on both the availability of signal molecules and the ability of the cells to sense them.	The effectiveness of PT, resulting from the preferential eradication of the wild-type or mutant sub-population, will be determined by the type of signaling molecules in the surrounding environment.

Results from the work by Mumford and Friman (2017) were omitted in this table because the experiments involved inter-species bacterial competition in addition to the different QS genotypes, hence, predicting the particular effect of QS in selection becomes more complex; nevertheless, we highlight the relevance of their findings, especially in the context of PT.

## Discussion

It is becoming increasingly clear that, among the broad array of bacteria-phage interactions, the link between QS and phages has a profound impact in the eco-evolutionary dynamics of microbial communities, therefore, it represents a relevant factor to consider in the context of infections and PT.

QS is a key topic in bacterial infection research since it regulates the production of virulence factors in several pathogens (Rutherford and Bassler, 2012; Castillo-Juárez et al., 2015). As these factors are typically used by the population to collectively change or take advantage of the surrounding environment, they are considered public goods. Cooperative behaviors require that many cells contribute to the pool of public goods to achieve efficiency. Nonetheless, this concerted effort is commonly prone to cheating by individuals that benefit from the pool of goods without contributing to it. The emergence of cheaters represents an interesting phenomenon in the context of infections. *P. aeruginosa* and *S. aureus* are examples of pathogens that use QS to control the expression of several virulence genes and for which mutations in QS genes (e.g., *lasR/rhlR* or *agr* genes, respectively) occur in infections (Hoffman et al., 2009; Paulander et al., 2012; Wang et al., 2018). The implications of this evolutionary process are unclear but this observation shows that QS mutants and isolates with wild-type QS genes occur during the course of infection.

Several QS-phage interactions reported so far show that cell-cell communication enhances bacterial survival against phages. Due to its nature, this anti-phage strategy confers enhanced phage tolerance, i.e., transient reduction in susceptibility to infection. The fact that similar relationships can be found in different bacterial species suggests, as other authors point out (Høyland-Kroghsbo, 2013; Høyland-Kroghsbo et al., 2017; Qin et al., 2017; Saucedo-Mora et al., 2017), that this anti-phage defense system is widely distributed. The phage defense attribute typically corresponds to a private good in the form of downregulation of the phage receptor, therefore, it can be expected that other mechanisms protecting the cell surface from interaction with phages can be regulated by QS as well. Importantly, the coupling of public goods production with private phage defense under the same regulation mechanism, QS, implies that phages can be key factors in stabilizing cooperation in mixed populations. In this type of interaction, the theory suggests preferential infection of QS mutants, which could have important implications in the context of PT if the target pathogen upregulates virulence genes through QS: selection of QS-proficient cells could cause an increase in virulence, either transient or stable depending on the efficacy of the defense system and hence the strength of the selection. Nevertheless, this interaction also opens the possibility of the combined use of PT and quorum quenching (QQ), which would increase the phage susceptibility of the population while decreasing virulence. As previously highlighted (Hoque, 2016), it is important to characterize the effect of QQ in the target pathogen to assess compatibility with PT, as in some cases, such as *V. cholerae*, QQ may promote increased virulence.

There are documented examples where QS-mediated phage defense represents a public good: proteases production by *V. cholera* (Hoque, 2016) and *V. anguillarum* (Tan et al., 2020). These extracellular enzymes have been proven to inactivate phage virions, potentially protecting both producer and cheater individuals in mixed populations. It is worth noting that predicting the competition outcome in an infection scenario becomes more difficult when considering the additional effect of public goods, as the final subpopulations proportion after PT treatment would depend on complex interactions; e.g. the strength of positive selection on the wild-type phenotype by lowering phage receptor expression and inactivating phages *via* proteases, combined with a negative selection factor resulting from the protease-mediated protection of QS mutants. This type of interactions, however, is particularly suitable to be investigated through experimental evolution and mathematical modeling approaches to simulate a series of scenarios (see Pérez-Velázquez et al., 2016; Cazares et al., 2020).

Intriguingly, the QS-phage interaction can have contrasting outcomes in terms of susceptibility to infection. Reports in *P. aeruginosa* and *E. coli* show that QS can promote phage susceptibility, although the mechanisms behind this effect are still unknown. This interaction type suggests that phages primarily target producer individuals, which could lead to selection of QS mutants. In a hypothetical infection scenario, this selection may decrease virulence if it is QS-regulated, and QQ may counteract PT since QS disruption would lead to reduction in phage susceptibility. It should be noted, however, that given the relevance of active QS systems in bacteria, other factors can play a role in stabilizing cooperation in the population, hence offsetting the selection pressure exerted by phages. One example is our work on selection of functional QS systems by *P. aeruginosa* temperate phages (Saucedo-Mora et al., 2017). We showed that D3112-like phages promote the relative fitness of QS-active cells compared to the signal blind *lasR rhlR* double mutants in mixed planktonic populations, thus counteracting exploitation by cheaters and selecting functional QS systems. In our study, phages replicated preferentially in the wild-type strain, predicting selection of the mutant population, however, we observed the opposite effect. We hypothesize that lysogeny was a key factor in the selection: since phages infect the wild-type isolates preferentially, the population of QS-proficient lysogens that become resistant to phage infection by homoimmunity grows rapidly, overtaking the cheaters subpopulation. The role of temperate phages in increasing competitiveness has been documented in *P. aeruginosa* (Davies et al., 2016). Additionally, there are multiple reports of QS-controlled factors (e.g., pyocyanin and rhamnolipids) promoting negative selection of the cheater’s subpopulation in this bacterial species (Wang et al., 2015; Castañeda-Tamez et al., 2018; García-Contreras et al., 2020).

It is worth noting that the findings on QS-phage interactions described above correspond to reports on both virulent and temperate phages (see Table 1, column 2). Historically, virulent phages have been preferred for therapy purposes over temperate viruses, mostly due to concerns on the latter potential to transfer virulence determinants and drive lysogenic conversion (Monteiro et al., 2018). This suggests that we should focus on the interactions with virulent phages, yet the QS-controlled anti-phage mechanisms reported so far seem to be generalist (e.g. downregulation of phage receptors and production of virion-inactivating extracellular proteases), and hence relevant to both phage types. Further research expanding susceptibility testing to a wider panel of phages and characterizing new anti-phage strategies will be necessary to confirm that this is the case. Moreover, recent advances in the fields of genomics and synthetic biology have led to a renewed interest in exploring the potential of temperate phage variants as therapeutic agents (see the review by Monteiro et al., 2018), underscoring the importance of using a broad variety of phages to characterize QS-driven shifts in susceptibility to infection.

New research shows that filamentous phages may represent public goods themselves, potentially affecting the sociobiology of their hosts. Apart from their role in immune system modulation, these phages of the *Inoviridae* family can promote virulence by enhancing the properties of biofilms toward increased adherence, and tolerance to antibiotics and desiccation (Secor et al., 2015). Future research will reveal whether strategies allowing cheating of filamentous phages exist, as expected from the cooperative nature of their production, and what bacterial mechanisms regulate the synthesis of this viral group.

The reports compiled here underline the wealth and diversity of bacteria-phage interactions, and their potential to affect the sociobiology of microbial populations, including in bacterial infections. The perspective presented is not an isolated effort, since the relevance of phage social interactions has been recently reviewed by Fernández et al. (2018) and Secor and Dandekar (2020). In particular, we underscore the importance of understanding the interplay between phages and bacterial communication strategies. It is clear that more research is necessary to assess how this relationship can impact PT efforts. For example, phage defense as a private good under the control of QS implies that phages help stabilizing cooperation, however, for this link to be evolutionary stable it should have a direct fitness benefit dependent on cell density (Schuster et al., 2017). This effect should then be tested through competition experiments in different conditions, especially in infection models, and using experimental evolution approaches. Not only the therapeutic phage and its target pathogen will exist in an infection, therefore, the fate of these populations will be shaped by their interaction with the surrounding environment, including the microbial community. Remarkably, elimination of a strain in the ecosystem could lead to the unintended selection of a different pathogen. In this regard, research shows that both QS and inter-species bacterial competition can indeed affect the outcome of phage infection in an *in vitro* PT model (Mumford and Friman, 2017). Furthermore, the QS-phage infection interlink can derive in distinct results depending on the bacterial pathogen, hence, a diversity of models needs to be investigated to make robust conclusions about this relationship. Finally, mathematical modeling can provide valuable insights into the outcome of complex interactions between multiple factors but it requires prior experimental knowledge of the system (Cazares et al., 2020); thus, we encourage others to integrate both approaches in any phage infection system aiming to recapitulate PT.

In concordance with others (see references in Table 1; Fernández et al., 2018; Secor and Dandekar, 2020), we highlight that discovery of phage interactions with various systems and discussion of their implications can be used to advance phage-based therapies. The study of phage-immune system interactions can lead to the development of vaccines protecting against bacterial infections (Sweere et al., 2019). Investigation of bacteria-phage interaction networks can be used to inform selection of phages, conditions, and order of application to optimize therapy (Wright et al., 2019). Likewise, our knowledge on QS-phage interactions and their role in bacterial cooperation can inform the combined use of PT and QQ, and aid in the selection of phages that are not affected by QS-driven defense mechanisms or that preferentially infect cells featuring QS-controlled virulent phenotypes. As PT steadily advances toward a systematic use in clinical environments, we emphasize that research in phage ecology and evolution is not desirable but critical for PT success, as it can support the design of strategies that minimize the risk of unintended side effects and optimize the stability and efficacy of therapy.

## Author Contributions

All authors listed have made a substantial, direct and intellectual contribution to the work, and approved it for publication.

### Conflict of Interest

The authors declare that the research was conducted in the absence of any commercial or financial relationships that could be construed as a potential conflict of interest.
